# Moral Judgement in Early Bilinguals: Language Dominance Influences Responses to Moral Dilemmas

**DOI:** 10.3389/fpsyg.2018.01070

**Published:** 2018-06-28

**Authors:** Galston Wong, Bee Chin Ng

**Affiliations:** ^1^Linguistics and Multilingual Studies, School of Humanities, Nanyang Technological University, Singapore, Singapore; ^2^Neurolinguistics and Cognitive Science Laboratory, Nanyang Technological University, Singapore, Singapore; ^3^Psychology, School of Social Sciences, Nanyang Technological University, Singapore, Singapore

**Keywords:** early bilinguals, language dominance, moral dilemmas, decision-making, emotion

## Abstract

The Foreign-Language effect (FLe) on morality describes how late bilinguals make different decisions on moral judgements, when presented in either their native or foreign language. However the relevance of this phenomenon to early bilinguals, where a language's “nativeness” is less distinct, is unknown. This study aims to verify the effect of early bilinguals' languages on their moral decisions and examine how language experience may influence these decisions. Eighty-six early English-Chinese bilinguals were asked to perform a moral dilemmas task consisting of personal and impersonal dilemmas, in either English or Mandarin Chinese. Information on language experience factors were also collected from the participants. Findings suggest that early bilinguals do show evidence of a language effect on their moral decisions, which is dependent on how dominant they are in the language. Particularly, the more dominant participants were in their tested language, the larger the difference between their personal and impersonal dilemma response choice. In light of these findings, the study discusses the need to re-examine how we conceptualize the FLe phenomenon and its implications on bilinguals' moral judgement. It also addresses the importance of treating bilingualism as multidimensional, rather than a unitary variable.

## Introduction

Would you sacrifice one life to save the lives of several others? Researchers in the field of moral psychology have studied this conundrum in order to understand individuals' motivations and the decision-making processes that occur in such dilemma scenarios. Previous studies have identified several factors that could influence a person's decision in the context of such dilemmas, many of which may be considered intuitive. For instance, people were more likely to sacrifice an individual life when the choice of action was impersonal rather than personal (e.g., Royzman and Baron, 2002; Greene et al., 2009; Kusev et al., 2016). People were also more likely to opt for sacrificing one life when the utilitarian outcomes of the decisions were more explicitly stated, indicating a framing effect of the textual description of the dilemma on moral decision-making (Kusev et al., 2016). Other contextual factors include characteristics of the individuals described in the scenarios, such as their race or group identity (e.g., Uhlmann et al., 2009; Swann et al., 2010). Specifically, these studies showed how people were more likely to make moral decisions that benefited individuals who were part of their in-group compared to the out-group.

What is perhaps less intuitive are the findings from recent studies showing how the language that the dilemmas are presented in may also influence our moral judgements (Costa et al., 2014b; Cipolletti et al., 2015; Geipel et al., 2015b). This has been attributed to the Foreign-Language effect (FLe) which describes how decision-making outcomes in a bilingual speaker can be influenced by the use of a native (L1) or foreign (L2) language (Keysar et al., 2012; Costa et al., 2014a). In the context of morality, the FLe predicts that bilingual individuals are more likely to make utilitarian choices when presented with a moral dilemma in their foreign language than in their native language (Cipolletti et al., 2015). A utilitarian decision refers to prioritizing the lives of the group majority over the one sacrificed individual and is considered as the more rational response.

Based on the premise of the FLe, it should be possible to predict that language would have negligible effects on moral decision-making if both languages were not considered foreign, that is, if both have similar status as first languages. We may expect individuals who are early bilinguals to exhibit similar patterns of moral choices regardless of the language medium, although this assumption has neither been discussed in-depth nor affirmed in the current literature. This could presumably be attributed to geographical limitations in recruiting a sample of such bilinguals from the community. To address this issue, the current study investigated how early English-Chinese bilinguals in Singapore responded to moral dilemmas presented to them in either English or Mandarin Chinese. Firstly, it aimed to explore the validity of the FLe on moral decision-making in the context of early bilinguals. It also aimed to investigate the extent to which differences in language background factors may have an influence on moral decision-making within a sample of early bilinguals.

One of the first studies to demonstrate the FLe phenomenon on moral decision-making was conducted by Costa et al. (2014b). In one of their experiments, English-Spanish bilinguals with either English or Spanish as their L1 were presented with the Footbridge and Trolley scenarios (for a detailed description, see Thomson, 1976) and asked to make a difficult decision of whether to sacrifice one individual to save five others from being killed by a runaway trolley. The Footbridge scenario is described as a personal dilemma, where the reader is presented the option to push an individual off a footbridge to save the group. Whereas the Trolley scenario is an impersonal dilemma, where one is asked to redirect the runaway trolley and indirectly sacrifice the individual. The findings from the study revealed that speakers were more likely to make utilitarian decisions in their respective L2, with a larger percentage of bilinguals responding “yes” to sacrificing an individual's life as compared to those who read it in their L1. This language effect on their moral decision-making was especially more pronounced in the personal Footbridge scenario than the impersonal Trolley scenario. These findings have also been replicated by other researchers who worked with different speakers from different countries and of different languages (e.g., Cipolletti et al., 2015; Geipel et al., 2015b; Chan's et al., 2016).

One explanation given for this more pronounced observation in the Footbridge scenario is that the personal dilemma elicits stronger emotional arousal than an impersonal dilemma (Costa et al., 2014b; Cipolletti et al., 2015), suggesting the important role of emotional intensity in eliciting the phenomenon. In addition, it has been suggested that a L2 tends to promote more systematic and rational decision-making than a L1 due to the weaker emotional weight and increased processing difficulty that is attributed to a later acquired language as compared to one acquired at an early age (Keysar et al., 2012; Costa et al., 2017). Indeed, past studies have shown that bilingual speakers tend to experience stronger emotional responses in an L1 as compared to an L2, both behaviorally (e.g., Anooshian and Hertel, 1994; Colbeck and Bowers, 2012) and physiologically (e.g., Harris et al., 2006).

In this regard, the dual-process theory appears most apt in explaining the findings from these studies on language and morality. According to its framework, individuals make decisions based on two processes, referred to as System 1 and System 2. System 1 is known as the automatic route in decision-making, which is usually attributed to implicit and emotional processing. System 2 on the other hand, referred to as the controlled processing route, is more commonly associated with explicit and logical decision-making (Stanovich and West, 2000; Reyna, 2004). A bilingual speaker's L1 is thus believed to activate the automatic route in decision-making processes, whereas the foreign language is more likely to activate the controlled route in System 2 due to attenuated emotionality and more deliberate objective processing (Costa et al., 2017).

However, there is still insufficient evidence to support the dual-process theory and its impact on emotional arousal on moral decision-making across different languages. While Geipel et al. (2015b) found that their participants had lower distress ratings in a L2 compared to L1, the measure was not significant as a mediating factor on the effect of language on their moral decisions on the Trolley and Footbridge tasks. In another study, Chan's et al. (2016) instead found positive correlations between participants' emotional arousal and utilitarian responses. Similarly, they did not observe a mediation effect of emotional arousal on participants' language and moral decisions. Hence, this study also sets out to verify these inconsistencies regarding the role of emotional arousal in mediating the effects of language on moral decision-making.

Additionally, it is still uncertain as to what aspects of a bilingual speaker's language experience contributes to the FLe observed in these moral decision-making studies, as current research has only studied the variable of bilingualism from a narrowly defined perspective. To the best of our knowledge, studies on moral dilemmas and bilingualism have focused only on late bilinguals who acquire their second language at a later age, some of which began from as late as 8–14 years (e.g., Costa et al., 2014b; Geipel et al., 2015b). As such, age of acquisition becomes a main indicator that differentiates between a speaker's two languages and presumably the driving factor of the FLe. However, age of acquisition alone does not succinctly encapsulate the various dimensions that make up a speaker's language background, nor does it encompass the various descriptors used to define the different possible types of bilingual speakers, such as balanced or dominant bilinguals (see Ng and Wigglesworth, 2007, p. 5–8). When Costa et al. (2014b) included an analysis of L2 proficiency, they found that the FLe effects were attenuated by higher L2 proficiency in the late bilinguals, Hence there is some evidence that proficiency is a variable in an L2 context. However, the issue of language dominance in the contexts of two L1 s, a widespread bilingual phenomenon, is still unexplored.

Here, we propose that early bilinguals would also show evidence of a language effect on their moral decisions, which is not determined by age of acquisition but by their language dominance. While early bilinguals may acquire both languages at an early age (usually before age of three), one simply cannot assume that the experiences for the two languages are necessarily comparable. Language experiences can differ across bilingual speakers and their exposures to each of the two languages are unlikely to be equivalent. Although both languages may be learned by an individual as first languages (L1), it can be argued that one language may still be considered more dominant over the other, due to the differing domains of daily language use by the speaker (Ng and Wigglesworth, 2007, p. 7). Indeed, differences in language experience have been found to influence aspects of emotionality across languages despite the assumption of the emotional closeness we assign to our L1 as compared to our L2 (Caldwell-Harris, 2014; Pavlenko, 2014, p. 280).

Degner et al. (2012) found that German and French bilinguals demonstrated significant automatic and emotional processing in a L2 affective priming task, but only when the speakers were exposed to and used it frequently. More recently, Kazanas and Altarriba (2016) have similarly proposed that language dominance plays an important role in influencing emotional resonance in a language and challenges the notion of a L1 being the bilingual speaker's most emotional language. This was evident in their study of Spanish-English bilinguals, where participants showed significantly faster responses in an emotional priming and lexical decision task in a L2, when it was their dominant language.

If the emotional distancing effect of a language is hypothesized to be an underlying factor influencing the moral FLe phenomenon, the more nuanced and insightful bilingual effects by more recent studies would suggest that early bilinguals may not have similar responses to moral dilemma scenarios regardless of the language medium.

The current study addresses this issue by examining how early bilinguals with varying degrees of language dominance respond on a set of personal and impersonal moral dilemmas. The aim is to observe the relationship between individuals' responses to the moral dilemmas and their language dominance. We also included a comparison of their within-subject differences in responses between a personal and impersonal choice dilemma. We predicted that, despite acquiring both languages at an early age, early bilinguals' moral decisions would be influenced by their language experience, such that this contrast difference would be larger for bilinguals who are exposed to the dilemma scenarios in their more dominant language than in their non-dominant language. In line with prior researchers' proposal, we also predicted that emotional arousal, measured by self-reported distress ratings, would have a negative correlation with their language dominance and dilemma responses.

## Methods

### Participants

Eighty-six university students of different majors were recruited from a University in Singapore (65 females, 21 males; age range = 19–28 years, *M*_age_ = 21.5, *SD* = 1.73). Singapore is a multilingual community where individual bilingualism is the norm. However, Singaporeans differ in their degree and nature of bilingualism which was controlled in this study.

All participants were native English-Chinese bilinguals who were born and raised in Singapore. A pre-study screening was conducted, in the form of a brief online self-report questionnaire, to ensure that recruited participants were early bilinguals. There is debate among researchers regarding the age of bilingual first language acquisition to be considered an early bilingual. While some researchers like De Houwer (1995) argue that early bilingual language acquisition should begin simultaneously from birth, others have proposed less stringent criteria that ranges from 12 months (Ng and Wigglesworth, p. 43) to three years old (McLaughlin, 1984). In the current paper, we included individuals who began acquiring both languages before the age of 3 years old.

The study procedure took ~30 min to complete and the students were given monetary compensation for their participation. Participants were randomly assigned to complete a moral dilemmas measure in either English or Mandarin Chinese, and followed by a language background questionnaire presented in English. A preliminary comparison showed no significant differences between the two task language conditions for participant characteristics and language background, *p* > 0.05 (Table 1).

**Table 1:** Participant characteristics and language background by task language condition.

**Characteristics**	**Task language condition**	
	**English**	**Mandarin Chinese**
	**(*n* = 43)**	**(*n* = 43)**
Age (in years)	21.23 (1.48)	21.77 (1.93)
Gender Ratio (Female, Male)	32, 11	33, 10
**AGE OF ACQUISITION (IN YEARS)**		
- English	0.63 (1.09)	0.40 (0.90)
- Mandarin Chinese	0.23 (0.65)	0.44 (0.93)
**Bilingual Language Profile Scores:**		
i Language History		
- English	93.3 (15.06)	98.23 (10.81)
- Mandarin Chinese	92.02 (10.17)	91.37 (14.32)
ii Language use (in Average Week)		
- English	33.53 (8.89)	35.65 (8.54)
- Mandarin Chinese	15.42 (8.96)	12.49 (8.53)
iii Language Proficiency
- English	21.09 (3.05)	21.77 (2.97)
- Mandarin Chinese	17.09 (5.07)	16.84 (3.71)
iv Language Attitudes		
- English	19.21 (4.49)	19.49 (4.38)
- Mandarin Chinese	18.26 (5.32)	17.44 (4.14)

*Language background was measured by the Bilingual Language Profile (Birdsong et al., 2012). Means and standard deviations are reported for each group condition, where applicable*.

### Materials and procedures

#### Moral dilemmas task

Ten English scenarios were selected for the current study, adapted from a list of moral dilemmas compiled in a study by Christensen et al. (2014). The scenarios were translated to Mandarin Chinese and cross verified by three native Mandarin Chinese speakers. The 10 scenarios can be categorized into five pairs of situations that differed based on the choice of action proposed, either personal or impersonal. These five sets of scenarios were the *Burning Building, Crying Baby, Organ Transplant, Shark Attack*, and *Trolley/Footbridge* dilemmas (Appendix A in Supplementary Material). Across all the scenarios, the death of the single individual can be avoided, if not sacrificed, and the number of lives that can be saved in the dilemmas range from five to eleven. This is to control for the possible confounds arising from outcome inevitability of the sacrificed individual and the size of the rescued group, respectively.

The task was conducted on a computer screen with the use of E-Prime 2.0 computer software (Schneider et al., 2002). A practice trial was first done to familiarize participants to the task before proceeding to the actual set of experiment scenarios. All 10 scenarios were presented randomly one at a time. For each scenario, participants would first read a paragraph description of the scenario, together with another paragraph detailing the possible choice of action to carry out and its consequence. Once participants had finished reading the first slide, they would proceed to the next slide by using the “spacebar” key. On this second slide, they were then presented with a paragraph which reiterated the given choice of action and asks the participants whether they would commit to it. Using the “1” to “7” numbered keys, participants would then respond to the question on a 7-point utilitarian scale (1 = definitely no/ 绝对不会 [jué duì bù huì], 7 = definitely yes/ 绝对会 [jué duì huì]). For all scenarios, the questions were framed in a utilitarian manner such that higher ratings on the scale reflected more willingness to sacrifice the individual in order to save the group majority. After inputting their response, a third slide would then be presented, asking participants to rate how distressing the scenario felt to them (“Thinking about the scenario I just read, I feel very troubled”/“想着刚读过的短文中的情况, 我感到很困扰” [xiǎngzhe gāng dúguò de duǎnwén zhong de qíngkuàng, wo gǎndào hěn kùnrǎo]), which was adopted from a similar measure used by Geipel et al. (2015b). This was also performed on a 7-point scale using the same numbered keys (1 = strongly disagree/非常不同意 [fēicháng bù tóngyì], 7 = strongly agree/非常同意 [fēicháng tóngyì]) and was used as a measure of the emotional arousal of the participants when reading the scenarios.

#### Bilingual language profile

Participants' language background for English and Mandarin Chinese was assessed using the Bilingual Language Profile (BLP; Birdsong et al., 2012), which was administered on a computer screen. The BLP is a self-reported assessment of a bilingual speaker's language experience and dominance in the 2 target languages. It assesses a bilingual's language background quantitatively and computes calculated scores for each language across 4 modules—*language history, use (in an average week), proficiency*, and *attitudes*. The maximum score obtainable for the 4 modules are 120, 50, 24, and 24, respectively. An overall language dominance score for each language is also calculated based on the summation of weighted scores from the 4 different modules, with a total possible score of 218. Both the English and Mandarin Chinese language dominance scores and the individual raw scores from the 4 modules were used in the present study's analyses.

### Data analysis

To test for the main effects of language and dilemma type for each of the five sets of scenario pairs, a 2 × 2 mixed-factor analysis was conducted to compare the utilitarian ratings with dilemma type (personal/impersonal) as a within-subject variable and language (English/Mandarin Chinese) as a between-subject variable. There were two rationales for this analysis step. One of them was to observe for any differences between the 2 language conditions in the participants' overall utilitarian response ratings. More importantly, this analysis would allow us to identify scenario pairings with significant differences in responses between a personal and impersonal dilemma type, which would then be retained for further analysis. While each of the selected scenario pairings may be distinguished as either a personal or impersonal dilemma type, the content of the two scenarios may still evoke similar impact and utilitarian responses. As such, these scenarios may not provide useful information for subsequent comparisons.

To examine the relationship between participants' language dominance and moral decisions, a correlational analysis was then conducted between their BLP language dominance and their mean scenario contrast scores, which was calculated as the average scenario contrast ratings across all the retained scenarios from the previous analyses. The contrast score for each scenario pair was calculated by subtracting the participants' personal utilitarian response rating from their impersonal response rating for the same dilemma scenario, which allowed us to obtain a within-subject measure for our study that would account for individual differences. The correlation was performed with all 86 participants, using the selected language dominance scores that corresponded to the participants' tested language condition in the moral dilemmas.

Lastly, the relationship between emotional intensity and participants' moral responses was also examined by correlational analyses. This was conducted between their self-reported emotional distress ratings with their utilitarian ratings within each of the scenarios, regardless of the task language condition.

## Results

### Utilitarian ratings across scenarios and language medium

**Figure 1:**
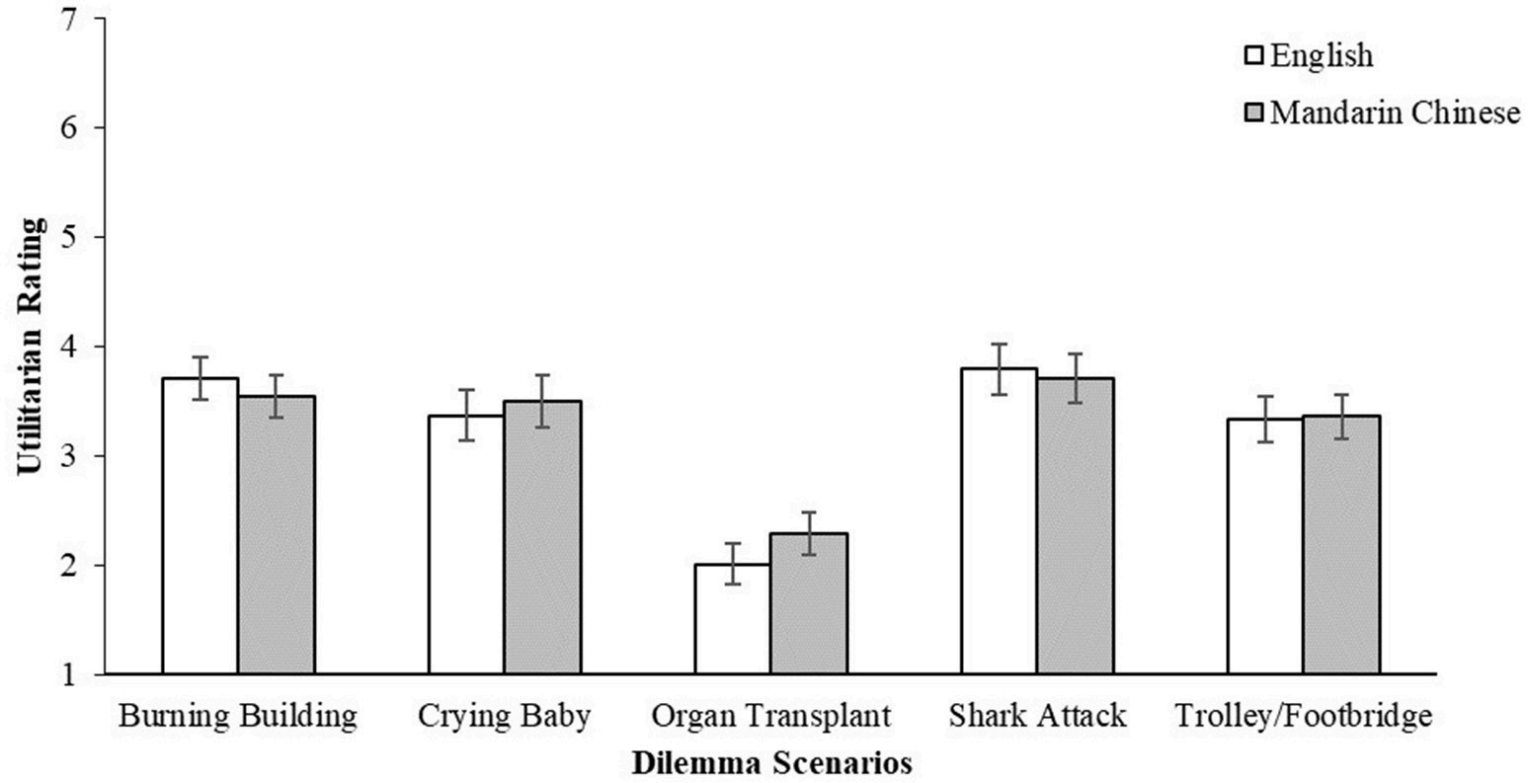
Utilitarian ratings (1 = definitely no, 7 = definitely yes) by dilemma scenarios and language (English/Mandarin Chinese). Error bars represent standard errors of the means.

**Figure 2:**
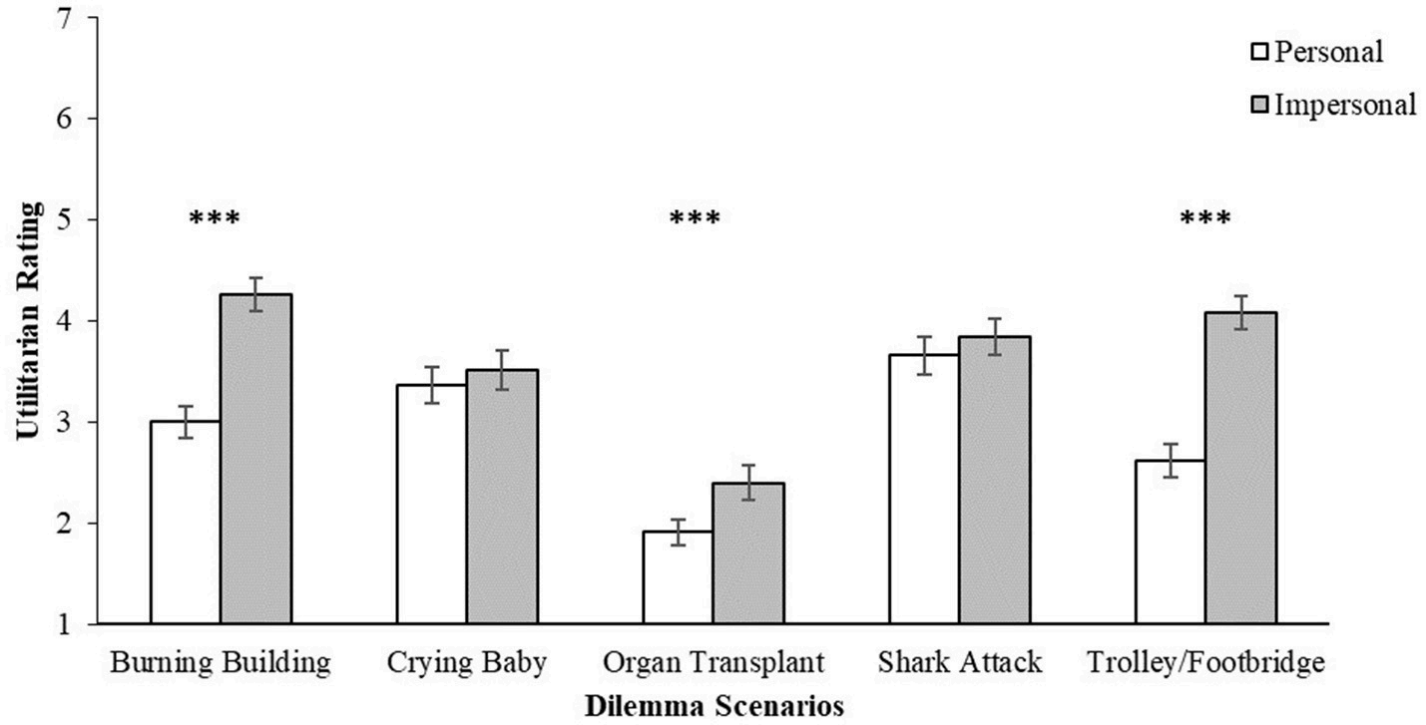
Utilitarian ratings (1 = definitely no, 7 = definitely yes) by dilemma scenarios and personal force (personal/impersonal). Error bars represent standard errors of the means. ***p < 0.001.

The mixed-factor analyses found no main effect of language across all five different scenarios on the participants' utilitarian ratings, *p* > 0.05, indicating that participants responded similarly across both the English and Mandarin Chinese versions of the task (Figure 1). A main effect of dilemma type was found for 3 out of 5 of the scenario pairs—*Burning Building* [*F*_(1, 84)_ = 54.3, *p* < 0.001, $\eta_{\text{p}}^{2}$ = 0.393]*, Organ Transplant* [*F*_(1, 84)_ = 11.8, *p* < 0.001, $\eta_{\text{p}}^{2}$ = 0.123] and *Trolley/Footbridge* [*F*_(1, 84)_ = 78.8, *p* < 0.001, $\eta_{\text{p}}^{2}$ = 0.484]—with the personal choice of action being rated significantly lower on the utilitarian scale compared to the impersonal choice (Figure 2). These three scenario pairs were retained for the subsequent analyses.

**Figure 3:**
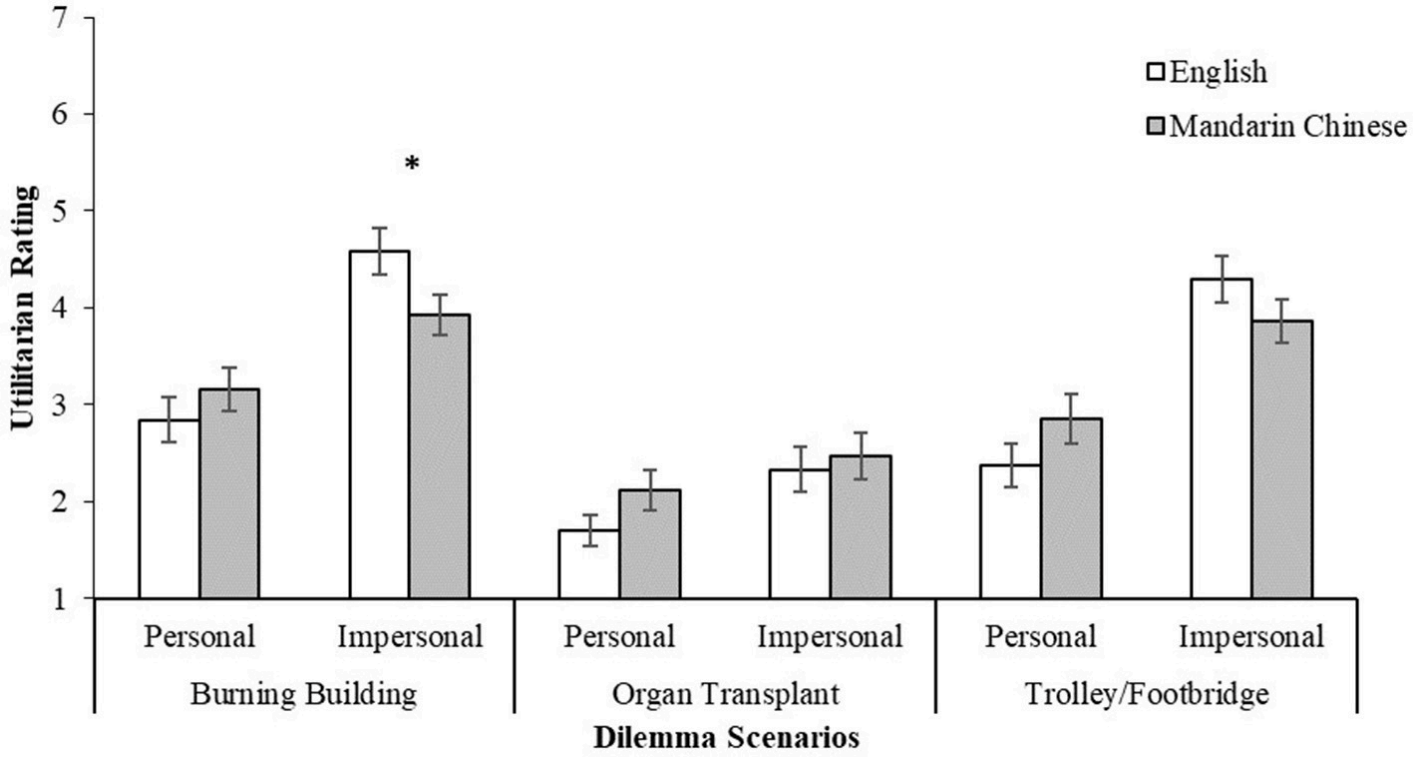
Utilitarian ratings (1 = definitely no, 7 = definitely yes) of the *Burning Building, Organ Transplant* and *Trolley/Footbridge* scenarios by personal force (personal/impersonal) and language (English/Mandarin Chinese). Error bars represent standard errors of the means. *p < 0.05.

Looking at the interaction effects for the three retained scenarios, a significant interaction was found for the *Burning Building* [*F*_(1, 84)_ = 8.21, *p* = 0.005, $\eta_{\text{p}}^{2}$ = 0.089] and *Trolley/Footbridge* scenarios [*F*_(1, 84)_ = 7.94, *p* = 0.006, $\eta_{\text{p}}^{2}$ = 0.086], but not the *Organ Transplant* scenario [*F*_(1, 84)_ = 0.965, *p* = 0.329, $\eta_{\text{p}}^{2}$ = 0.011]. In both significant cases, the difference between the utilitarian response for the personal and impersonal dilemmas were larger in English, as compared to the Mandarin Chinese condition (Figure 3). Further *post-hoc* analyses were also conducted on the three scenario pairs to observe for the simple effects of the language medium on the utilitarian ratings. With the exception of the *Burning Building–Impersonal* dilemma having a marginal significance (*p* = 0.042), there were no significant differences in the participants' utilitarian ratings between the English and Mandarin Chinese versions of the dilemmas.

### Scenario contrast scores and language dominance

Consistent with our predictions, a mild positive correlation was found between the overall language dominance of participants and their mean scenario contrast scores, *r*_(86)_ = 0.377, *p* < 0.001. This suggests some evidence of a language influence on participants' moral choices, such that the more dominant they were in the language of their tested condition, the larger the average contrast between the personal and impersonal choice of action in a given scenario. Furthermore, correlational analyses were also conducted to compare the relationship between the mean contrast scores and the four different modules of BLP, using an adjusted *p*-value of 0.0125 for the four comparisons. Three out of four of the BLP modules—use, proficiency and attitudes—also showed similar positive correlations with the mean contrast scores (Table 2). Despite all four of the modules showing significant intercorrelations with each other to varying extents (Appendix B in Supplementary Material), language history was not correlated with the mean contrast scores.

**Table 2:** Pearson's correlation coefficients between mean scenario contrast scores and language dominance module scores across of participants (*N* = 86).

**Language dominance modules**	**Mean Contrast Scores**	
	***r***	***p***
- History	0.043	0.692
- Usage	0.355	<0.001^*^
- Proficiency	0.291	0.006^*^
- Attitudes	0.361	<0.001^*^

* *p < 0.0125. The language dominance score selected for each participant (English/Mandarin Chinese) corresponded with their task language condition*.

A follow-up analysis was also carried out to further validate the significance of language dominance on the participants' moral decisions by comparing between groups with different degrees of language dominance on the mean scenario scores. Four language dominance groups were obtained by dividing the participants into four different percentile ranges. They were categorized as either low (below 25th percentile), mid-low (25th-−50th percentile), mid-high (50th-−75th percentile) or high language dominance (above 75th percentile) groups with respect to the current study sample. A brief overview of the group characteristics is reported in Table 3. As a larger proportion of our participants generally had higher language dominance in English than in Mandarin Chinese, it was not possible to equate all 4 groups in terms of task language condition.

**Table 3:** Overview of language dominance groups' participant composition and language dominance scores (corresponding to task language condition), with means and standard deviations reported.

**Characteristics**	**Language Dominance Group**			
	**Low (*n* = 21)**	**Mid-Low (*n* = 22)**	**Mid-High (*n* = 22)**	**High (*n* = 21)**
Task language (English: Chinese)	5:16	5:17	13:9	20:1
Language dominance	111.63 (13.29)	139.56 (7.25)	161.91 (7.41)	193.60 (8.69)

**Figure 4:**
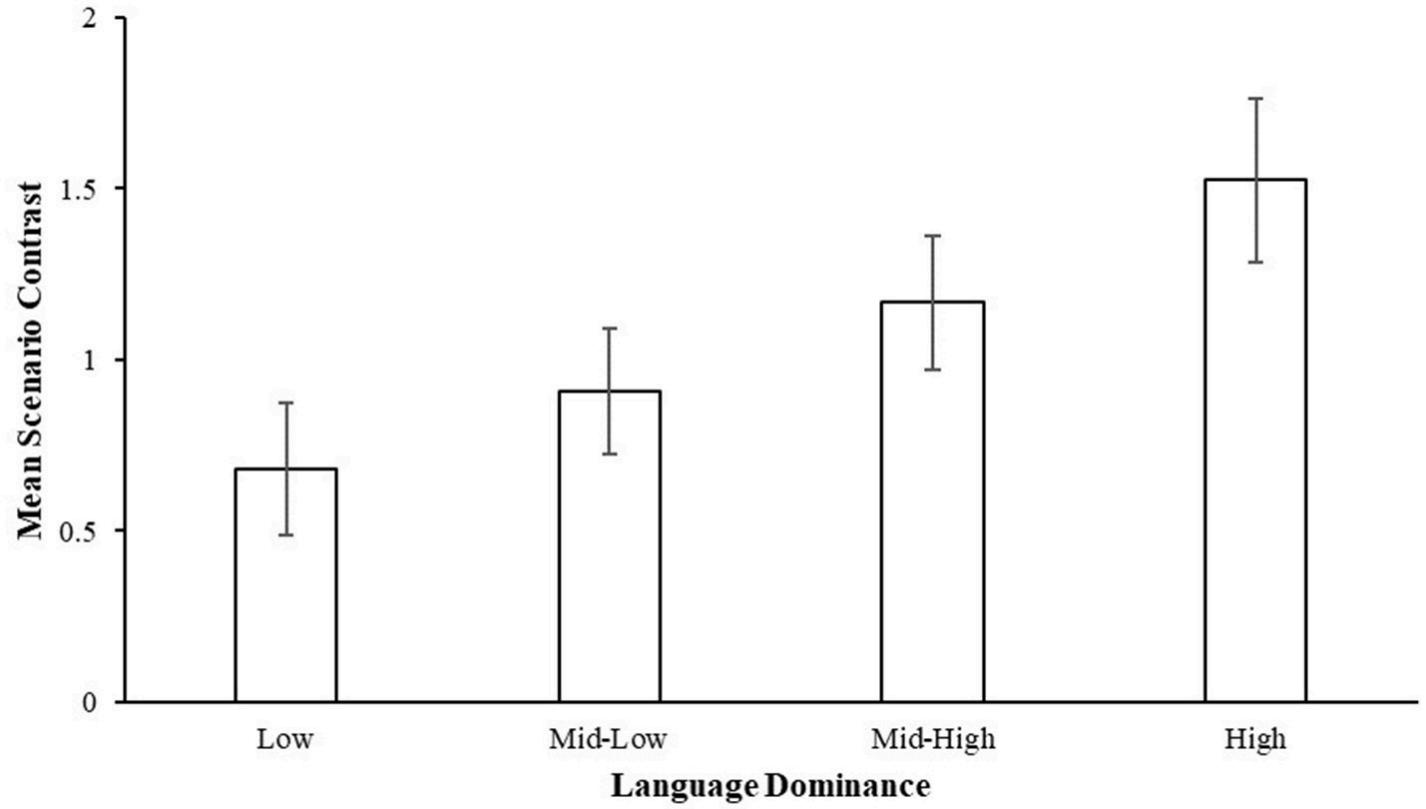
Mean scenario contrast scores by language dominance groups. A higher score indicates a larger average difference in utilitarian ratings between an impersonal minus personal choice of action in the same scenario type. Error bars represent standard errors of the group means.

An independent one-way ANOVA indicated a significant difference between the groups' mean scenario contrast scores, *F*_(3, 82)_ = 3.10, *p* = 0.031, η^2^ = 0.102 (Figure 4). *Post-hoc* tests with a Bonferroni correction revealed that participants in the high language dominance group (*M* = 1.52, *SD* = 1.09) had a larger mean contrast score between personal and impersonal choice of action as compared to those in the low language dominance group (*M* = 0.68, *SD* = 0.88), *p* = 0.029. There were no significant differences found with comparisons made with the other two dominance groups, mid-low (*M* = 0.91, *SD* = 0.89) and mid-high (*M* = 1.17, *SD* = 0.91).

### Language dominance, distress, and utilitarian ratings

Lastly, correlational analyses revealed weak to mild positive correlations between participants' self-reported emotional distress and utilitarian ratings for four of the scenarios—*Burning Building-Personal*, both *Organ Transplant*, and the *Footbridge* (Personal) scenario (Table 4). This was inconsistent with our initial expectations of a negative correlational relationship between the 2 variables as suggested in prior studies. Instead, we observed that the larger utilitarian response ratings were, the higher the emotional feeling of distress was self-reported. In addition, there were no correlations between participants' language dominance scores and their emotional distress ratings across all 6 of the dilemmas, *p* > 0.05.

**Table 4:** Correlation coefficients between utilitarian ratings and self-reported emotional distress intensity within each dilemma scenario across all participants (*N* = 86).

**Dilemma Scenarios**	**Pearson's Correlation Coefficient**	
	***r***	***p***
**BURNING BUILDING**		
- Personal	0.316	0.003^*^
- Impersonal	0.004	0.97
**ORGAN TRANSPLANT**		
- Personal	0.376	<0.001^*^
- Impersonal	0.277	0.01^*^
**TROLLEY/FOOTBRIDGE**		
- Personal	0.261	0.015^*^
- Impersonal	0.209	0.053

*Significant p-values are marked in asterisk if below 0.05*.

## Discussion

The present study investigated the relationship between early English-Chinese bilinguals' moral judgement and their language dominance. This was achieved by examining the bilinguals' performance on a set of moral dilemmas and the BLP. Initially, we found no differences in utilitarian ratings between the English and the Mandarin Chinese version of the moral dilemma task across the different scenarios. Similarly, there was no consistent evidence that suggested any differences when we observed for the simple effects of language across 3 pairs of scenarios, where there was a significant difference between the personal and impersonal choice dilemmas. From this perspective, it may be inferred that the findings would support the moral FLe, as our early bilinguals did not show influences of language on their moral decisions, unlike prior studies on late bilinguals who acquire a second language later in life. However, this was not the case when we examined if there was a correlation between their language dominance and their moral decision-making responses. Here, we conducted these tests using the contrast scores calculated as the difference between the impersonal and personal choice dilemmas from the same scenario type. The rationale for this was to obtain a within-subject measure that would allow for a comparison between participants' own responses and their language dominance. Consistent with our predictions, we found significant relationships between the speakers' language dominance and moral judgement such that the more dominant an individual was in their tested language, the larger the difference in that individual's mean dilemma contrast score. Lastly, contrary to our predictions, emotional arousal in our study showed a positive relation with the utilitarian ratings across a number of dilemmas and was not related to our participants' language dominance.

### Impact of language experience on moral judgement

A consideration brought up by the findings is determining how language experience contributes to the language differences observed in the FLe. The findings in this study indicates that prior conclusions of the FLe cannot easily be applied to early bilinguals and that bilingualism may be more accurately addressed as a complex multidimensional variable. In fact, what we see from the differences observed among early bilinguals may be more accurately referred to as a language dominance effect (LDe). Based on the correlations between individual BLP module scores with the scenario contrast scores, we observe that the frequency of language use and proficiency contributed to the LDe in influencing the early bilinguals' responses on the moral dilemmas. This would be consistent with evidence from the late bilinguals in Costa et al. (2014b), showing that higher L2 proficiency attenuated the FLe on their decisions for the *Trolley/Footbridge* scenarios. On the other hand, our participants' language history did not correlate with the contrast scores, which brings into question whether the FLe phenomenon is dependent on age of L2 acquisition. As language dominance variables tend to be linked, it is common to observe intercorrelations among them (Caldwell-Harris, 2014), which was also evident in our sample of English-Chinese bilinguals. Individuals who acquire a L2 language later in life may subsequently have lower proficiency and frequency of use as compared to their L1. As such, it is difficult to ascertain whether late bilinguals in past studies (see Costa et al., 2014b; Cipolletti et al., 2015; Geipel et al., 2015b) made more utilitarian responses due to influences from late language acquisition or more specifically its associated language experience factors, such as lower language proficiency.

Interestingly, individuals' attitudes toward the language and the culture were related to their moral choice responses. The more positive they felt toward the language, the less likely they were to accept a utilitarian response for a personal action as compared to an impersonal one. A possible explanation may be attributed to the interplay between language use, identity and psychological distancing. Research has shown that individuals' language can be a significant reflection of his identity and cultural affiliations (Tong et al., 1999; Pavlenko, 2004; Luna et al., 2008), with some multi-linguals even reporting personality and cognitive changes when shifting from one language to another (Dewaele and Nakano, 2013). Norton (2000) appealed to the concept of *language investment* to describe how language learners are not only acquiring a new language in the process, but are also actively reshaping their own self-identity and perceptions of society. We suggest that psychological distancing may occur from priming of different identities and affiliations associated with using different languages. A speaker who feels less attached to a language in his linguistic repertoire may also feel a sense of divestment from the subsequent decisions that he makes in that language. This may result individuals feeling less responsible for their actions, and reflected in their increased willingness in making the aversive decision of sacrificing a person's life. Though at this point, more research would be required to affirm the link between language attitudes and moral decision-making.

### Revisiting the FLe and dual-process theory

The current findings also points to a need to reassess the FLe, specifically regarding how we should define the outcomes of the FLe in the context of moral judgement and decision-making. While earlier studies concluded that a foreign language leads one to make more utilitarian decisions in moral dilemmas (Costa et al., 2014b; Cipolletti et al., 2015), this cannot be inferred from our current study. Our findings could be more accurately interpreted as given the same scenario; individuals are less likely to endorse the use of a personal choice of force, compared to an impersonal choice. What we observe here is that the permissibility of a difficult personal choice becomes more acceptable and easier to make in a non-dominant language. This is consistent with Corey et al. (2017) argument that the FLe may be attributed to a “reduced aversion to the action associated with saving the larger number of people,” rather than priming a more rational mentality.

Likewise, Geipel et al. (2015a, 2016) found an FLe effect despite the fact that in their study there were no requirements for participants to make a decision based on utility. Instead, the participants were asked to judge the “moral goodness” of an action carried out in a hypothetical scenario. In their studies, transgressions and negative intentions were judged less severely in a speaker's L2 than in a L1. Thus, defining the moral FLe based on the premise of utility and rationality does not sufficiently describe the outcomes observed in these studies and the current findings. In fact, studies by Hayakawa et al. (2017) have even suggested that the FLe affects moral decision-making by influencing System 1 processing and reducing deontological responses, rather than eliciting a more rational mindset.

This brings into question the appropriateness of applying a framework of utilitarianism with the dual-process theory to understanding morality in bilingual speakers. While our decisions may be affected by the use of a foreign or non-dominant language, they do not necessarily equate to better or logical choices and are dependent on the situation (Hayakawa et al., 2016). In this case, choosing to save more lives does not, by default, make it the correct or rational choice. We believe this to be especially true in the context of most moral situations, where the concept of a “right” decision is more ambiguous and also likely shaped by shared cultural norms and values (Cook, 1999, p. 8–9). Several studies have shown that individuals who endorsed more utilitarian decisions on moral dilemmas also tended to score higher on measures of antisocial personality traits (e.g., psychopathy and life meaninglessness), which many may consider as negative and immoral (Bartels and Pizarro, 2011; Pletti et al., 2017). As such, the applicability of conventional normative ethics and principles in understanding the FLe, at least in the context of moral choice dilemmas, may be contentious.

### The role of emotional arousal

Contrary to our predictions, we found evidence suggesting that stronger emotional arousal toward a moral dilemma corresponded with greater utilitarian responses, especially in the personal choice dilemma. However, it should be noted that studies reporting on emotional arousal and its' mediating influence on bilingual individuals' moral responses have not been definitive, even suggesting the necessity of an alternative explanation for the findings (Geipel et al., 2015b). In fact, our findings are consistent with Chan's et al. (2016), who also found similar positive correlations in their sample of Chinese-English bilinguals from Hong Kong. This may reflect similar underlying cultural influences that are present among the two groups of Chinese speakers which could have affected their moral decisions. Although further research would be required to verify if the affective reactions to the moral dilemmas vary across culture and how it interacts with language. At the same time, it also suggests that while emotional arousal may be related to language dominance (Kazanas and Altarriba, 2016) and moral decision-making (Greene et al., 2001), it may not necessarily be the direct mediating factor between the two as initially believed.

A limitation may be that our current measure of emotional arousal was unable to capture the emotional aspect of the experiment that was intended. This would not be uncommon, given that the validity of self-reported behavioral measures in affective research can be inconsistent, though not necessarily unimportant (Mauss and Robinson, 2009). As the measure was introduced at the end of each scenario presentation, the participants may have rated their emotional responses to making a difficult decision instead, i.e., a high distress rating may reflect a participant's discomfort in his final decision to sacrifice the individual in the dilemma.

## Conclusion

We aimed to explore how the moral FLe would apply to a group of early English-Chinese bilinguals in their decision-making based on 10 dilemma scenarios, either in English or Mandarin Chinese. We also aimed to determine if there was a relationship between language dominance and the bilinguals' responses to these scenarios. The present study is the first to look at the use of language dominance as a variable in elaborating the FLe as well as attempting to examine the relationship between variables of language experience and moral decision-making in a quantitative manner. The findings from our study provides evidence that language dominance may have a potential influence on bilinguals' moral judgements and at the same time, may be a more suitable descriptor in accounting for the FLe and LDe phenomenon. The present study also highlights the complex role of emotional arousal and its relation to language dominance and morality. Given the inconsistent findings observed in studying emotional arousal and utilitarian response patterns, alternative explanations may be needed to account for the role of emotional arousal in the moral FLe and LDe phenomenon. Lastly, as the nature of the present study was largely exploratory, we suggest that future research address and affirm whether language dominance affects bilinguals' moral judgements by using discretely defined groups of participants (e.g., high and low dominant bilingual speakers).

## Ethics statement

This study was carried out in accordance with the recommendations of the university's NTU-IRB ethics committee with written informed consent from all subjects. All subjects gave written informed consent in accordance with the Declaration of Helsinki. The protocol was approved by the NTU-IRB ethics committee.

## Author contributions

GW and BN conceived and designed the experiments. GW conducted the experiment and analyzed the results. GW and BN wrote the paper.

### Conflict of interest statement

The authors declare that the research was conducted in the absence of any commercial or financial relationships that could be construed as a potential conflict of interest.
