# Supplementary material for: Moral Judgement in Early Bilinguals: Language Dominance Influences Responses to Moral Dilemmas
Source: Front Psychol. 2018 Jun 28;9:1070. doi: 10.3389/fpsyg.2018.01070 (PMC6032433; doi:10.3389/fpsyg.2018.01070)
Supplement: Supplementary file 1 [file Table_1.DOCX]

**Appendix A: List of moral dilemmas**

| **Dilemma** | **English** |  | **Mandarin Chinese** |
| --- | --- | --- | --- |
| *Burning Building – Personal* | You and five other people are trapped in a burning building. There is only one emergency exit through which all of you could escape, but it is blocked by burning debris. Another injured person is about to crawl through a hole at the bottom of the exit door. You and the other five people do not have time to do the same.  If you use the injured person to break down the debris, you will be able to escape. Doing this will certainly kill him, but it will save you and the five people behind you.  Do you break down the blockage by using the injured person so you and the five other people can escape? |  | 你和其他五人被困在一栋失火的楼里。这栋楼里只有一个逃生口可以让你们全部人逃出，但它被着火的瓦片挡住了。一个受伤的人正打算从逃生门底下的洞钻出去，但他若这么做，你和其他五人就没有时间做跟他一样的事。  如果你利用那个受伤的人把瓦片打破，你们将有机会逃出。这么做一定会杀了他，但却可救出你自己和在你后面的五个人。  你会利用那位受伤的人来打破障碍物以让你自己和其他五个人逃生吗？ |
| *Burning Building – Impersonal* | You and five other people are trapped in a burning building. There is only one emergency exit through which all of you could escape, but it is blocked by burning debris. Another injured person is about to crawl through a hole at the bottom of the exit door. You and the other five people do not have time to do the same.  If you activate the emergency system, it will eliminate oxygen from the hallway which will put out the fire. However, it will leave the injured person without air. This will kill him, but you and the five people behind you will be saved.  Do you put out the fire by activating the emergency system, which will leave the injured without air, so you and the five other people can escape? |  | 你和其他五人被困在一栋失火的楼里。这栋楼里只有一个逃生口可以让你们全部人逃出，但它被着火的瓦片挡住了。一个受伤的人正打算从逃生门底下的洞钻出去，但他若这么做，你和其他五人就没有时间做跟他一样的事。  如果你启动紧急系统，这将会削减在走廊的氧气并停止火势。然而，这会使那位受伤的人没有氧气。这将会杀了他，但你和其他五人将会得救。  你会为了扑灭火势而启动紧急系统，致使那位受伤的人没有空气，让你和其他五人可逃生吗？ |
| *Crying Baby – Personal* | Enemy soldiers have taken over your village and will kill all civilians above the age of two. You and ten neighbours are hiding in two rooms of the cellar of a large house. You can hear the voices of soldiers who have come to search the house for valuables. Your baby begins to cry loudly. The crying will attract the attention of the soldiers, who will spare your baby’s life, but will kill you and the other refugees in both rooms.  If you put your hand over its mouth, the crying will be absorbed, but your baby will not be able to breathe. This will kill it, but it will save you and the other ten neighbours.  Do you absorb the crying by putting your hand over your baby’s mouth, which will leave it without air, so the soldiers won’t find you and the ten neighbours? |  | 敌军已占领你的村庄并即将杀掉所有两岁以上的市民。你和其他十位邻居躲在一栋大房子里的地下室中的两个房间里。你可以听见已经进屋搜寻值钱物品的士兵们的声音。你的婴儿这时开始大哭。哭声将会引来士兵的注意，他们会放过你的婴儿，但会把你和其他躲在两个房间里的邻居们杀掉。  如果你用手捂住婴儿的嘴巴，婴儿的哭声将会被盖住，但它便会无法呼吸。这将会杀了它，但却会救了你自己和其他十个邻居。  你会为了掩盖哭声而用手捂住婴儿的嘴巴吗？这样的话，婴儿将无法呼吸，但士兵们将不会找到你及其他十个邻居。 |
| *Crying Baby – Impersonal* | Enemy soldiers have taken over your village and will kill all civilians above the age of two. You and ten neighbors are hiding in two rooms of the cellar of a large house. You can hear the voices of soldiers who have come to search the house for valuables. Your baby begins to cry loudly. The crying will attract the attention of the soldiers, who will spare your baby’s life, but will kill you and the other refugees in both rooms.  If you activate a noisy boiler it will cushion the crying, but it will become uncomfortably hot. The heat will be mortal for your baby, but it will save you and the ten neighbors.  Do you cushion the crying by activating the noisy boiler which will suffocate your baby, so they won’t find you and the ten neighbors? |  | 敌军已占领你的村庄并即将杀掉所有两岁以上的市民。你和其他十位邻居躲在一栋大房子里的地下室中的两个房间里。你可以听见已经进屋搜寻值钱物品的士兵们的声音。你的婴儿这时开始大哭。哭声将会引来士兵的注意，他们会放过你的婴儿，但会把你和其他躲在两个房间里的邻居们杀掉。  如果你启动一个很吵的热水器，这将会掩盖住婴儿的哭声，但同时也会将你们置身于不舒服的热度。高温将会热死你的婴儿，但会救了你及其他十名邻居。  你会启动那个非常吵但可以盖住哭声的热水器吗？这样的话，你的婴儿将会被热死，但士兵们将不会找到你和其他十个邻居。 |
| *Organ Transplant – Personal* | You are a doctor. You have five patients who will all die if they don’t get an immediate organ transplant. Another patient is just being rushed to the hospital after a serious car accident. This person is critically injured, but you can probably save him by means of a long and complicated operation.  If you secretly cut his carotid artery during surgery it will cause his death and you will have the organs for the other five patients. This will kill him, but save the other five patients.  Do you obtain the organs cutting the carotid artery of the accident victim, so you can undertake the transplantations for the other five patients? |  | 你是一位医生。你有五个病人，如果他们没有马上得到器官移植的话，他们将会全部死掉。有一位病人因为历经了一场严重的车祸才刚刚被推进医院。这名患者的伤势非常严重，但如果你执行一个长时间并复杂的手术，你应该可以救了他。  如果你在手术期间偷偷地割他的颈动脉，他将会死去而你便可以把他的器官移植给你的其他五位病人。这将会杀死他但会救其他五位病人。  你会为了得到那些器官而割这位车祸受害者的颈动脉吗？这样的话，你就可以获得他的器官去救其他五位病人。 |
| *Organ Transplant – Impersonal* | You are a doctor. You have five patients who will all die if they don’t get an immediate organ transplant. Another patient is just being rushed to the hospital after a serious car accident. This person is critically injured. During the long and complicated surgery, you notice that a nurse is about to inject the wrong medication, which will be lethal in this case.  If you let the nurse inject the wrong medication the accident victim will die and you will have the organs for the other five patients. This will kill this patient, but will save the other five patients.  Do you obtain the organs by letting the nurse inject the wrong medication, so you can undertake the transplantations for the other five patients? |  | 你是一位医生。你有五个病人，如果他们没有马上得到器官移植的话，他们将会全部死掉。有一位病人因为历经了一场严重的车祸才刚刚被推进医院。这名患者的伤势非常严重。当你为他执行一个长时间并复杂的手术时，你注意到一位护士即将注射错误的药物，那将会致命。  如果你让这名护士为这名车祸的受害者注入错误的药物，你将会有器官可以移植到那五位病人。这将会杀死这个病人但会救其他五位病人。  你会为了得到器官而让那名护士注射错误的药物到车祸受害者的身上吗？这样的话，你就可以获得他的器官去救其他五位病人。 |
| *Shark Attack – Personal* | You and ten divers are part of an U.N. team who is deactivating anti-ship mines from World War II. One team member has hurt himself and the blood in the water has attracted several sharks. You have an underwater rifle but only one harpoon and there are many sharks. The bleeding diver is swimming towards the last protective cage and will reach it before you and the others. The sharks, following the blood, are coming too close for you and the other divers to escape.  If you shoot at the injured diver this will kill him and the sharks will stop to eat him, but you and the nine divers will be saved.  Do you let the sharks eat the injured diver by shooting at him, so you and the other nine divers can reach the protective cage? |  | 你和十位潜水员是U.N.队的成员，你们正在解除第二次世界大战所留下来的防船水雷。其中一位队员受伤了，血在水里吸引了几只鲨鱼。你有一支水底来福枪，可是只有一枚子弹，无法抵抗那么多鲨鱼。流血的潜水员正游向最后一个保护笼，他将会比你和其他人更快到达那个笼子。追随血腥的鲨鱼即将迅速地跟上你们，使你们无法逃脱。  如果你开枪杀了那位受伤的潜水员，鲨鱼们便会游向他并吃了他，但你和其他九位潜水员能因此得救。  你会为了让鲨鱼去吃那位受伤的潜水员而开枪射杀他吗？如此一来，你和其他九位潜水员便能安全抵达保护笼。 |
| *Shark Attack – Impersonal* | You and ten divers are part of an U.N. team who is deactivating anti-ship mines from World War II. One team member has hurt himself and the blood in the water has attracted several sharks. You have an underwater rifle, but only one harpoon and there are many sharks. The bleeding diver is swimming towards the last protective cage and will reach it before you and the others. The sharks, following the blood, are coming too close for you and the other divers to escape.  If you detonate one of the mines by means of a remote control, it will kill the injured diver and the sharks will stop to eat him. Then, you and the nine divers will be saved.  Do you let the sharks eat the injured diver by detonating the mine next to him with a remote control, so you and the other nine divers can reach the protective cage? |  | 你和十位潜水员是U.N.队的成员，你们正在解除第二次世界大战所留下来的防船水雷。其中一位队员受伤了，血在水里吸引了几只鲨鱼。你有一支水底来福枪，可是只有一枚子弹，无法抵抗那么多鲨鱼。流血的潜水员正游向最后一个保护笼，他将会比你和其他人更快到达那个笼子。追随血腥的鲨鱼即将迅速地跟上你们，使你们无法逃脱。  如果你借着遥控器引爆其中一枚水雷，这将会杀死那名受伤的潜水员并且使鲨鱼停止追你们而转去吃他，你和其他九位潜水员便会获救。  你会为了让鲨鱼去吃那位受伤的潜水员而用遥控器引爆在他旁边的水雷吗？如此一来，你和其他九位潜水员便能安全抵达保护笼。 |
| *Footbridge (Personal)* | A runaway trolley is speeding down the tracks towards five workmen who will be killed if the trolley continues on its present course. You are standing next to the tracks, but you are too far away to warn them. Next to you there is a very large stranger.  If you push the large stranger onto the tracks, the trolley will slide off the tracks and won’t continue its course towards the workmen. This will kill the stranger, but you will save the five workmen.  Do you cause the trolley to derail by pushing the stranger onto the tracks, so the trolley does not reach the five workmen? |  | 一辆失速的电车正沿着轨道朝着有五位工人的方向冲去，如果不停止这辆电车的话，这五位工人将会被撞死。你站在轨道旁边，但因离五位工人太远所以无法警告他们。你旁边站着一位高大的陌生人。  如果你把这位高大的陌生人推到轨道上的话，电车将会滑出轨道而不会继续朝着五位工人的方向前进。这将会杀了那位陌生人，但你会救了五位工人。  你会把那位陌生人推向轨道而使电车脱离轨道吗？如果这样的话，电车将不会到达五位工人。 |
| *Trolley (Impersonal)* | A runaway trolley is speeding down the tracks towards five workmen who will be killed if the trolley continues on its present course. You are standing next to the tracks, but you are too far away to warn them. Next to you there is a control switch that can redirect the trolley onto a different track, where only one workman is working.  If you flip the control switch it will divert the trolley onto the track with one workman and will not continue its course towards the five workmen. This will kill this workman, but it will save the other five.  Do you divert the trolley by flipping the control switch, which will cause the trolley to run over one workman, so the trolley does not reach the five workmen? |  | 一辆失速的电车正沿着轨道朝着有五位工人的方向冲去，如果不停止这辆电车的话，这五位工人将会被杀死。你站在轨道旁，但离五位工人太远了所以无法警告他们。你旁边有一个可以重新引导电车到另一条轨道的开关，而那条轨道上只有一位工人在工作。  如果你转动开关的话，电车将会转向有一位工人的轨道而不会继续驶向有五位工人的轨道。这样将会杀了那位工人，但会救了其他五位工人。  你会为了改变电车行驶的方向而转动那个控制开关吗？如此一来，电车将会辗过一位工人，并不会到达其他五位工人。 |
|  |  |  |  |
