# Supplementary material for: Moral Judgement in Early Bilinguals: Language Dominance Influences Responses to Moral Dilemmas
Source: Front Psychol. 2018 Jun 28;9:1070. doi: 10.3389/fpsyg.2018.01070 (PMC6032433; doi:10.3389/fpsyg.2018.01070)
Supplement: Supplementary file 2 [file Table_2.DOCX]

**Appendix B: Intercorrelations among Bilingual Language Profile (BLP) Modules**

|  | History | Use | Proficiency | Attitudes |
| --- | --- | --- | --- | --- |
| History | - | .31^**^ | .37^***^ | .28^**^ |
| Use | - | - | .63^***^ | .44^***^ |
| Proficiency | - | - | - | .52^***^ |
| Attitudes | - | - | - | - |
| ^**^ *p* < .01, ^***^ *p* < .001 (two-tailed). | | | | |
